# Supplementary material for: Hypervolume Niche Dynamics and Global Invasion Risk of Phenacoccus solenopsis under Climate Change
Source: Insects. 2024 Apr 5;15(4):250. doi: 10.3390/insects15040250 (PMC11050190; doi:10.3390/insects15040250)
Supplement: Supplementary file 1 [file insects-15-00250-s001.zip › insects-2940450-supplementary.pdf]

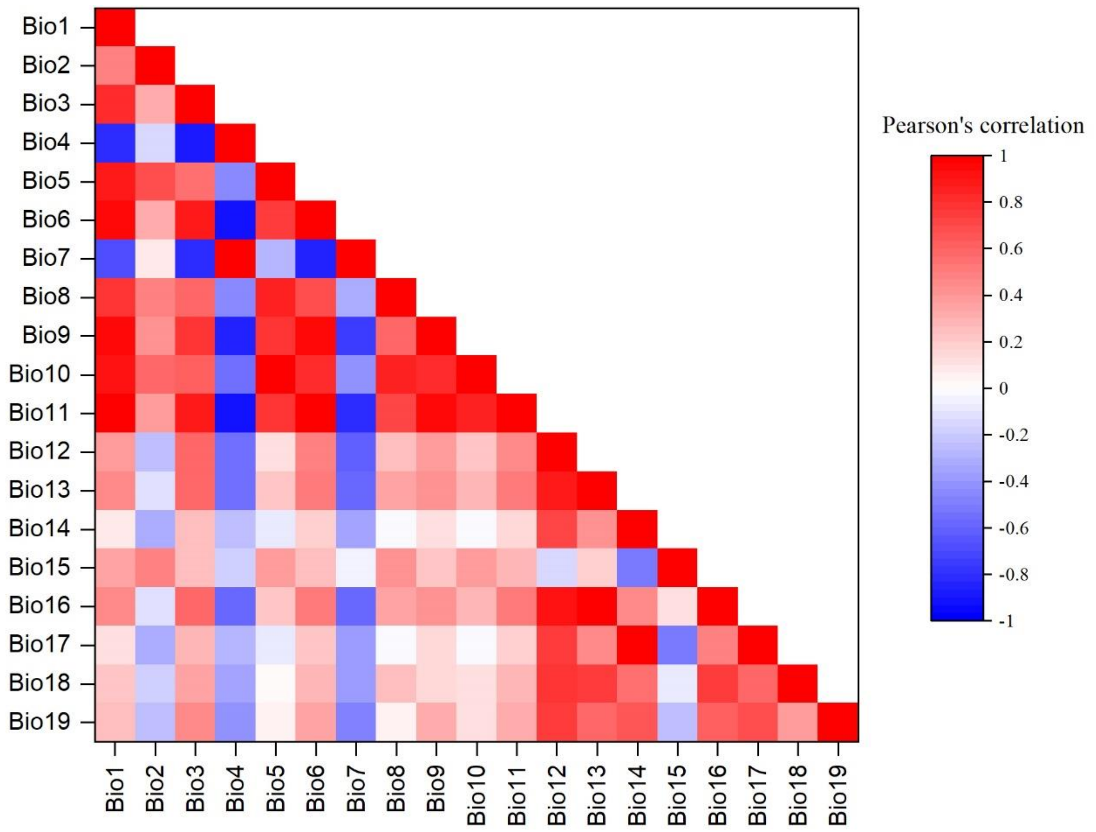

Figure S1 Pearson’s correlation of 19 bioclimatic variables

Table S1 The eigenvectors of 19 bioclimatic variables in the first five principal components

Variables	PC1	PC2	PC3	PC4	PC5
Bio1	0.010	-0.022	0.007	0.009	-0.003
Bio2	-0.001	-0.003	0.003	0.003	-0.003
Bio3	0.017	-0.026	-0.001	0.004	-0.005
Bio4	-0.468	0.876	0.008	0.076	0.034
Bio5	0.003	-0.011	0.006	0.014	0.002
Bio6	0.016	-0.031	0.002	0.006	0.000
Bio7	-0.013	0.020	0.004	0.008	0.001
Bio8	0.005	-0.008	0.016	0.008	-0.011
Bio9	0.013	-0.032	-0.006	0.012	0.008
Bio10	0.004	-0.011	0.006	0.011	-0.001
Bio11	0.015	-0.032	0.006	0.009	-0.001
Bio12	0.772	0.409	-0.073	-0.167	0.341
Bio13	0.118	0.043	0.158	0.270	-0.051
Bio14	0.023	0.025	-0.067	-0.126	0.044
Bio15	-0.002	-0.026	0.084	0.133	-0.068
Bio16	0.321	0.126	0.375	0.633	-0.098
Bio17	0.081	0.083	-0.222	-0.406	0.142
Bio18	0.166	0.153	0.449	-0.481	-0.717
Bio19	0.179	0.103	-0.752	0.249	-0.574
